# What can qualitative research do for randomised controlled trials? A systematic mapping review

**DOI:** 10.1186/1745-6215-14-S1-O52

**Published:** 2013-11-29

**Authors:** Alicia O'Cathain, Kate Thomas, Sarah Drabble, Anne Rudolph, Jenny Hewison

**Affiliations:** 1 University of Sheffield, Sheffield, UK; 2 University of Leeds, Leeds, UK

## Objective

To develop an empirically-based framework of the aspects of randomised controlled trials addressed by qualitative research.

## Design

Systematic mapping review of qualitative research undertaken with randomised controlled trials and published in peer-reviewed journals.

## Data sources

Medline, Premedline, Embase, The Cochrane Library, Health Technology Assessment, PsychINFO, CINAHL, British Nursing Index, Social Sciences Citation Index and ASSIA.

## Eligibility criteria

Articles reporting qualitative research undertaken with trials published between 2008 and September 2010; health research; reported in English.

## Results

296 articles met the inclusion criteria. They had a wide international authorship. Articles focused on 22 aspects of the trial within five broad categories. Some articles focused on more than one aspect of the trial, totalling 356 examples. The qualitative research focused on the intervention being trialled (71%, 254/356); the design, process and conduct of the trial (15%, 54/356); the outcomes of the trial (1%, 5/356); the measures used in the trial (3%, 10/356); and the target condition for the trial (9%, 33/356). A minority of the qualitative research was undertaken at the pre-trial stage (28%, 82/296).

## Conclusions

A large amount of qualitative research undertaken with specific trials has been published, addressing a wide range of aspects of trials, with the potential to improve the endeavour of generating evidence of effectiveness of health interventions. Researchers can increase the impact of this work on trials by undertaking more of it at the pre-trial stage and being explicit within their articles about the learning for trials and evidence-based practice.

